# Plasma proteomic evidence for increased β-amyloid pathology after SARS-CoV-2 infection

**DOI:** 10.1038/s41591-024-03426-4

**Published:** 2025-01-30

**Authors:** Eugene P. Duff, Henrik Zetterberg, Amanda Heslegrave, Abbas Dehghan, Paul Elliott, Naomi Allen, Heiko Runz, Rhiannon Laban, Elena Veleva, Christopher D. Whelan, Benjamin B. Sun, Paul M. Matthews

**Affiliations:** 1 UK Dementia Research Institute Centre at Imperial College London, London, UK; 2 Department of Brain Sciences, Faculty of Medicine, Imperial College London, London, UK; 3 Department of Psychiatry and Neurochemistry, Institute of Neuroscience and Physiology, Sahlgrenska Academy, University of Gothenburg, Mölndal, Sweden; 4 Clinical Neurochemistry Laboratory, Sahlgrenska University Hospital, Mölndal, Sweden; 5 Department of Neurodegenerative Disease, UCL Institute of Neurology, University College London, London, UK; 6 UK Dementia Research Institute Centre at UCL, London, UK; 7 Hong Kong Center for Neurodegenerative Diseases, Hong Kong, China; 8 Wisconsin Alzheimer’s Disease Research Center, University of Wisconsin School of Medicine and Public Health, Madison, WI USA; 9 Department of Epidemiology and Biostatistics, Faculty of Medicine, Imperial College London, London, UK; 10 MRC Centre for Environment and Health, Imperial College London, London, UK; 11 British Heart Foundation Centre of Research Excellence, Imperial College London, London, UK; 12 National Institute for Health Research Biomedical Research Centre, Imperial College London, London, UK; 13 Health Data Research UK at Imperial College London, London, UK; 14 Nuffield Department of Population Health, University of Oxford, Oxford, UK; 15 UK Biobank, Stockport, UK; 16 Translational Sciences, Biogen, Cambridge, MA USA; 17 Neuroscience Data Science, Janssen Research & Development, Cambridge, MA USA; 18 The Rosalind Franklin Institute, Didcot, UK

**Keywords:** Alzheimer's disease, Predictive markers, Viral infection

## Abstract

Previous studies have suggested that systemic viral infections may increase risks of dementia. Whether this holds true for severe acute respiratory syndrome coronavirus 2 (SARS-CoV-2) virus infections is unknown. Determining this is important for anticipating the potential future incidence of dementia. To begin to do this, we measured plasma biomarkers linked to Alzheimer’s disease pathology in the UK Biobank before and after serology-confirmed SARS-CoV-2 infections. SARS-CoV-2 infection was associated with biomarkers associated with β-amyloid pathology: reduced plasma Aβ42:Aβ40 ratio and, in more vulnerable participants, lower plasma Aβ42 and higher plasma pTau-181. The plasma biomarker changes were greater in participants who had been hospitalized with COVID-19 or had reported hypertension previously. We showed that the changes in biomarkers were linked to brain structural imaging patterns associated with Alzheimer’s disease, lower cognitive test scores and poorer overall health evaluations. Our data from this post hoc case–control matched study thus provide observational biomarker evidence that SARS-CoV-2 infection can be associated with greater brain β-amyloid pathology in older adults. While these results do not establish causality, they suggest that SARS-CoV-2 (and possibly other systemic inflammatory diseases) may increase the risk of future Alzheimer’s disease.

## Abstract (web summary)

Blood biomarkers in a middle-aged population suggest that SARS-CoV-2 infection is associated with greater brain β-amyloid plaque accumulation.

## Main

Exposures to infectious diseases in early life and adulthood have been linked to increased risk for neurodegenerative and other systemic disease. Viral encephalitis or meningitis, influenza, pneumonia and viral intestinal infections have been linked to risk for Alzheimer’s disease (AD)^1–3^, and vaccinations to prevent these diseases may have protective effects^4^. A variety of mechanisms could underlie these effects, including tissue injury during the acute phase of infections or chronic secondary central or peripheral inflammatory processes^5–7^. However, large-scale, harmonized, prospective observations in human populations are required to allow common factors that predispose to both infection and disease to be discriminated from indirect associations between infection and dementia.

The recent COVID-19 pandemic provided a common viral exposure to large populations over well-defined periods. Although the virus is not neurotrophic^8^, impairment of cognition and brain structural changes have been reported as sequelae of severe acute respiratory syndrome coronavirus 2 (SARS‑CoV‑2) infections, even among people who did not require hospitalization or experience long COVID^9–11^. SARS-CoV-2 initiates a systemic inflammatory response, which persists in many patients beyond the acute phase and increases susceptibility to several systemic diseases. Initial evidence for raised dementia rates in vulnerable populations following serious COVID have been reported^12–14^. The COVID pandemic thus could provide a ‘natural experiment’ for testing whether systemic infection can initiate or potentiate brain pathology associated with AD. The global scale of the pandemic and aging populations make a better understanding of the potential impact of COVID-19 and other infections on the prevalence of future dementias a public health priority^9,10^.

Recently developed cerebrospinal fluid (CSF) and plasma proteomic biomarkers can identify pathology associated with dementia years in advance of symptoms or a clinical diagnosis and provide sensitive outcome measures for a study of early dementia-related pathology^15–18^. The best-characterized proteomic biomarkers associated with pathology linked to AD are decreases in β-amyloid-(1-42) (Aβ42) and its ratio with β-amyloid-1-40 (Aβ42:Aβ40), and increases in phosphorylated tau (pTau), all of which have been associated with brain β-amyloid pathology^15,19,20^. Levels of neurofilament light (NFL; a marker of neuronal injury) and glial fibrillary acidic protein (GFAP; associated with astrocyte activation) also can change with AD^18,21,22^. Decreases in CSF Aβ42 may precede the diagnosis of AD by up to 18 years, with later changes in the Aβ42:Aβ40 ratio, pTau, NfL and GFAP^17,18^. Plasma measures now have diagnostic accuracies approaching those in CSF and thus provide the opportunity to assess presymptomatic populations for evidence of the effects of SARS-CoV-2 on brain pathology that may be related to future AD risk^16,19,23^.

Dementia-related biomarker signatures have been reported in patients with acute or severe COVID-19 (refs. ^14,24,25^). However, to robustly test for relationships between SARS-CoV-2 infection and biomarkers of AD-related pathology requires longitudinal study designs of mild-to-moderate infections that are unconfounded by factors associated with severe illness. We were able to conduct an observational study incorporating these elements. We took advantage of the longitudinal blood sampling and clinical data acquisition in the UK Biobank in combination with that study’s surveillance for SARS-CoV-2 exposure serology during the early stage of the UK COVID-19 pandemic (2020–2021). We report results from 1,252 UK Biobank participants tested for changes in AD-related plasma proteomic biomarkers after SARS-CoV-2 infection including a demographically matched control group sampled over the same interval. This longitudinal, post hoc matched case–control UK Biobank dataset permitted the assessment of potential confounders including aging, baseline differences in genetic backgrounds, sex and health status between cases and controls. SARS-CoV-2-infection-related changes in the plasma biomarkers could be linked to health, cognitive, imaging and further proteomic data.

## Results

### Study population

**Fig. 1:**
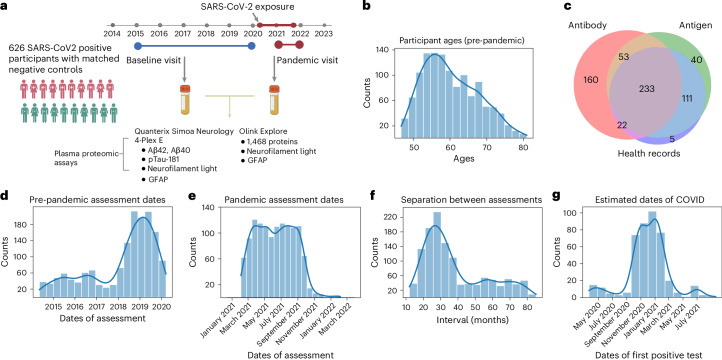
Study overview. **a**, Experimental design. Protein concentrations were assayed from plasma samples acquired from the UK Biobank imaging assessment visits, the second of which was specifically recruited for the study of COVID-19. **b**, Distribution of participant ages at the pandemic assessment. **c**, Sources of evidence for case selection. Antibody, home-based lateral-flow SARS-CoV-2 antibody test; Antigen, PCR antigen (swab) test; Health records, GP and/or hospital records. **d**, Distribution of pre-pandemic assessment visit dates. **e**, Distribution of pandemic assessment visit dates. **f**, Distribution of intervals between assessments. **g**, Estimated dates of COVID symptoms (from participants with antigen test results). Figure created with BioRender.com.

We studied plasma proteomic biomarkers previously associated with AD, as well as proteins from a general proteomic panel, brain images and other health data that were made available from 1,252 UK Biobank participants who had been recruited for the UK Biobank COVID-19 study^9^ (626 matched case–control pairs, 331 female; Fig. 1a). All participants had taken part in an imaging assessment session before the pandemic onset (baseline, May 2014 to March 2020). Participants were 46–80 years of age at this session (Fig. 1b). Individuals from these assessments were invited for the COVID-19 imaging study and were determined to have previously had evidence for SARS-CoV-2 infection by at least one of the following: home-based lateral-flow SARS-CoV-2 antibody test, PCR antigen (swab) test, general practitioner (GP) records or public health records (Methods and Fig. 1c). COVID-19 symptoms in cases were mostly mild, but 20 participants were hospitalized for COVID-19 or its complications. Cases were matched individually to eligible controls (based on age, sex, ethnicity, location and date of imaging assessment) who had no record of confirmed or suspected SARS-CoV-2 infection at the time of the repeat assessment. The COVID imaging assessments occurred during the UK COVID-19 pandemic (February 2021 to February 2022). Intervals between the pre-pandemic and pandemic assessment sessions ranged from 12 months to 82 months, matched for case–control pairs (Fig.1d–f). Blood samples for plasma proteomics were obtained at both imaging assessment sessions.

We characterized cases and controls for potential comorbidities and other factors (Table 1). Some characteristics differed between case and control groups, reflecting lifestyle factors that could increase the likelihood of early infection: more were employed before the pandemic (395 versus 364), more cases (*n* = 18) than controls (*n* = 6) identified as ‘key workers’ during the pandemic (*P* = 0.023), household sizes were larger on average (*P* = 0.007) and cases were more active on average (*P* = 0.002). Cases were slightly heavier (1.4 kg) than controls (*P* = 0.028). Other AD comorbidities did not show statistically significant differences across cases and controls. Slightly higher numbers of cases reported smoking or a history of hypertension.

**Table 1:** Comparisons of baseline characteristics of cases and controls

	Baseline characteristics	Cases	Matched controls	*P* values	*n*
Demographics	Age (baseline)	60.21 (7.41)	60.23 (7.41)	0.324	624
	Age (pandemic)	63.45 (7.02)	63.46 (7.02)	0.456	624
	Sex	0.47 (0.50)	0.47 (0.50)	1.000	624
	Height	170.34 (8.82)	169.89 (9.08)	0.222	622
	Weight	77.06 (15.02)	75.63 (14.54)	0.028	593
	Ethnicity (white)	0.93 (0.26)	0.92 (0.28)	0.437	624
Genetics	*APOE-ε3ε4*	123	110	0.869	423
	*APOE-ε4ε4*	11	9	0.788	423
	*APOE-ε3ε2*	73	62	0.547	423
Health measure	Hip-to-waist ratio	1.16 (0.12)	1.16 (0.12)	0.831	595
	BMI	26.56 (4.21)	26.23 (4.17)	0.110	593
	BP (systolic)	137.23 (19.22)	137.26 (18.58)	0.984	424
	BP (diastolic)	79.26 (11.04)	79.33 (10.80)	0.625	424
	Hand grip	32.01 (10.26)	31.75 (10.42)	0.691	587
	Chest wheeze	90	94	0.872	607
	Health self-rating	3.02 (0.62)	3.09 (0.64)	0.075	618
	Glomerular filtration rate	88.12 (10.52)	86.62 (10.87)	0.071	225
	Age-related vulnerability	0.981 (1.08)	0.982 (1.08))	0.789	624
Life status	Alcohol intake frequency	3.19 (1.34)	3.18 (1.32)	0.966	618
	Smoker	26	18	0.283	619
	Deprivation	16.50 (11.79)	15.56 (12.14)	0.230	590
	Income	2.83 (1.70)	2.82 (1.75)	0.893	620
	Number in household	2.46 (1.19)	2.30 (1.09)	0.007	620
	Key worker	18	6	0.023	624
	Employed	395	364	0.061	614
	Social isolation	34	48	0.137	624
	Moderate activity (min d^−1^)	63.25 (79.19)	52.29 (55.34)	0.002	503
	Vigorous activity (min d^−1^)	42.92 (41.70)	42.36 (39.26)	0.576	293
Comorbidity	Type 2 diabetes	17	15	0.858	624
	Heart condition	108	100	0.595	624
	Obesity	113	107	0.710	624
	Hypertension	145	127	0.244	624
	Depression	26	20	0.453	624
	IBS	39	34	0.629	624
	COPD	4	3	1.000	624
	Emphysema	3	4	1.000	624
Medications	Blood pressure medication	53	46	0.530	624
	Cholesterol medication	46	44	0.913	624
	Diabetes medications	3	3	1.000	624
	COVID vaccinated	384	388	0.351	286
Cognition	Cognitive ability score	32.84 (7.27)	32.94 (7.59)	0.967	407
Neuroimaging	AD neuroimaging phenotype	−2.29 (0.66)	−2.30 (0.66)	0.799	590

Cases and matched controls columns show mean values for the group (when standard errors shown), otherwise counts of participants with stated characteristic. *P* values reflect paired *t*-tests across matched case and control pairs (uncorrected). UK Biobank fields and derivation of measures can be found in Supplementary Table 1 and Methods. IBS, irritable bowel syndrome; BMI, body mass index; BP, blood pressure; BP medication, prescribed antihypertensive medications; COPD, chronic obstructive pulmonary disease.

**Extended Data Fig. 1:**
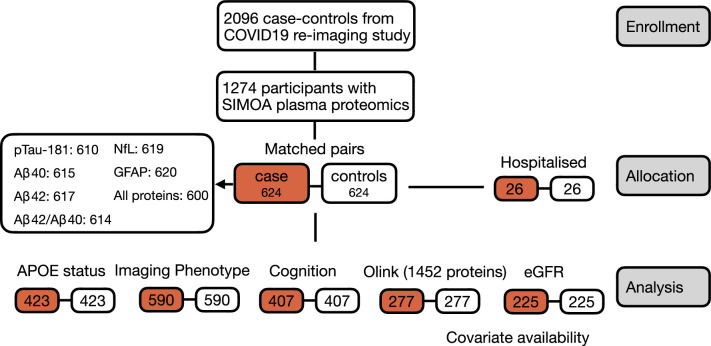
CONSORT Diagram. Schematic summary of data availability for the study. Primary analyses focus on 624 individually matched cases and controls from the COVID19 imaging repeat study with SIMOA plasma proteomic data. Full details of participant selection and control matching for the COVID study can be found at https://biobank.ndph.ox.ac.uk/ukb/ukb/docs/casecontrol_covidimaging.pdf.

Single-molecule array (Simoa) ultrasensitive measures of plasma biomarkers previously associated with AD (Aβ42, Aβ40, pTau-181, GFAP and NfL) were made for all blood samples (Methods). A complete set of longitudinal data was available for 600 cases and 600 matched controls. The remaining 26 matched pairs were missing data from one or more assays. Olink antibody-based proximity extension assay proteomic measurements of concentrations of 1,452 proteins were available for 277 of the case–control pairs^26^. Other variables were available in varying numbers of participants (Extended Data Fig. 1).

### The plasma Aβ42:Aβ40 ratio is reduced after SARS-CoV-2 infection

To test for effects of SARS-CoV-2 infection on biomarkers previously associated with AD, we assessed whether infection status affected longitudinal changes in their concentrations. In addition to the plasma concentrations, we also assessed the Aβ42:Aβ40 ratio^18,20^. We fit linear models describing each participant’s pandemic-session biomarker levels in terms of their baseline, pre-pandemic levels, time interval between the two sample acquisitions, age, sex and their individual case (serology positive, after SARS-CoV-2 infection) or control (serology negative, no evidence of previous SARS-CoV-2 infection) status (Methods). These models therefore describe changes in the plasma biomarker levels between assessments, with the case–control regressor identifying differences in these changes associated specifically with SARS-CoV-2 infection. Further models assessed the associations of a variety of comorbidities and other factors with biomarker levels at baseline and longitudinally (Methods and Supplementary Table 1).

**Fig. 2:**
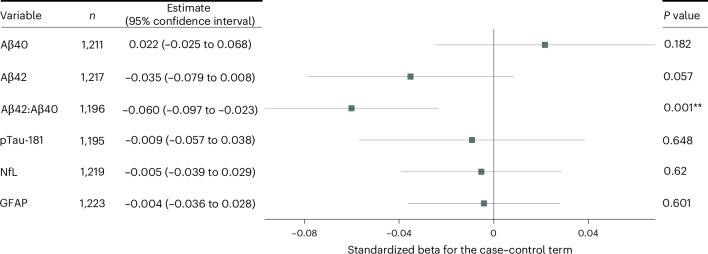
Standardized regression model parameter estimates for the case–control term in each of the AD protein change models. The parameters represent the SARS-CoV-2-infection-associated effect on change in protein levels between pre-pandemic and pandemic assessment sessions. Aβ42:Aβ40 ratios showed significant relative reductions in individuals with SARS-CoV-2. *P* values correspond to one-sided *t*-tests for differences corresponding to previously reported associations with AD. Full model fits are in Supplementary Tables 2 and 3 (basic and extended models). **FDR significant.

The average plasma concentrations of Aβ42, the Aβ42:Aβ40 ratio and pTau-181 decreased between baseline pre-pandemic and follow-up assessments during the pandemic for all participants (cases and controls), and plasma concentrations of Aβ40, NfL and GFAP increased (all *P* < 0.0001, paired *t*-tests). Statistical testing of the case–control model parameters found that SARS-CoV-2 infection was associated with a significantly greater reduction in the Aβ42:Aβ40 ratio (2% drop from baseline, false discovery rate (FDR) significant, **P = 0.0006; Fig. 2 and Supplementary Table 2). These results were maintained when an extended model including potential confounders was used (Supplementary Table 3). The effect size and statistical significance for the change in the Aβ42:Aβ40 ratio were maintained when cases identified by the less accurate lateral-flow antibody tests were excluded (case–control standardized beta = −0.701 (lateral flow excluded) versus −0.0601 (all participants)). The estimated effect of SARS-CoV-2 on the Aβ42:Aβ40 ratio was comparable to the effect of 4 years of aging (−0.5% change in the Aβ42:Aβ40 ratio per year of age, estimated at baseline) or around half the average effect of heterozygosity for *APOE-ε4* (*APOE-ε3ε4* participants showed a 3.9% lower Aβ42:Aβ40 ratio relative to *APOE-ε3* homozygous in the baseline assessment sessions). Other comparable comorbidities of AD did not show statistically significant associations with the Aβ42:Aβ40 ratio in this dataset. Greater reductions in the plasma Aβ42:Aβ40 ratio among cases were associated with a greater severity of symptomatic infections: cases who were hospitalized with COVID-19 showed over twice the magnitude of reduction relative to non-hospitalized cases (5.5% versus 2.0%).

### Changes in vulnerable participants

**Fig. 3:**
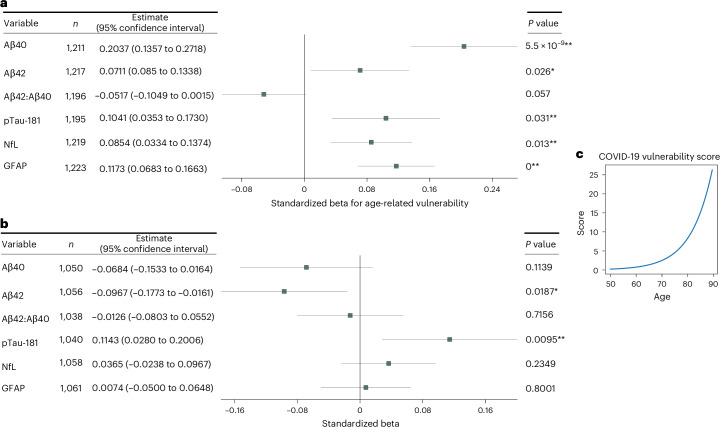
Standardized regression model parameter estimates for models including age-dependent vulnerability terms in each of the protein biomarker change models. **a**, Parameters for the age-dependent vulnerability term. **b**, Parameters for the age-dependent vulnerability term interaction with case–control status. **c**, Age-dependent vulnerability term weightings. *P* values correspond to one-sided *t*-tests for differences corresponding to previously reported associations with AD. Full model fits are in Supplementary Tables 4 and 5 (basic and extended models). *P < 0.05; **FDR significant.

Participants who were older or had a previous lifestyle or health conditions that are risk factors for neurodegenerative disease may be more vulnerable to infection-related neurodegenerative pathology. Age is a key risk factor both for AD and for the severity of COVID-19, but the relationships between age and risk are nonlinear; older people are more vulnerable to severe disease symptoms^27^. To test for specific effects of SARS-CoV-2 on plasma protein level changes in these more vulnerable, older study participants, we used regressors reflecting an age-related vulnerability score derived from observed associations between age and severe neurological outcomes for COVID-19 (Fig. 3c)^27^. This score has previously been used to analyze SARS-CoV-2 changes in brain MRI measures^9^. We assessed models that included this score (for cases and controls) and its interaction with case–control status to assess whether age-related vulnerability predicted changes in AD proteins between assessments with or without dependence on SARS-CoV-2 infection status (Methods).

**Fig. 4:**
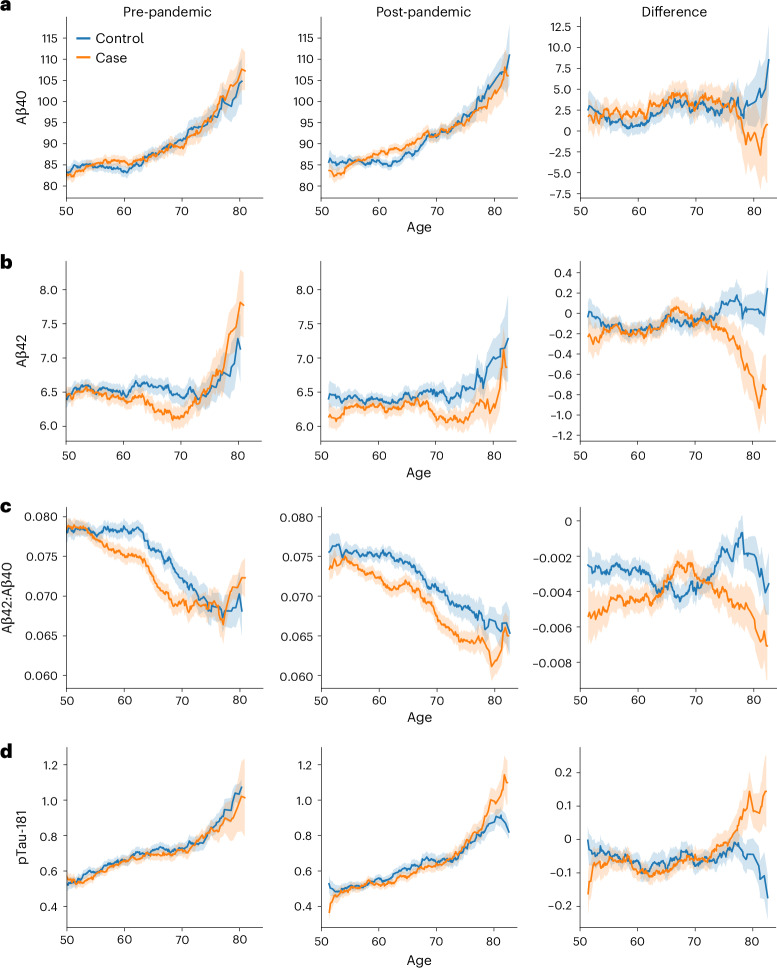
Time plots of plasma protein levels (pg ml^−1^) of SARS-CoV-2 cases. **a**, Aβ40. **b**, Aβ42. **c**, Aβ42:Aβ40 ratio. **d**, pTau-181. Plots show the 7 year rolling means for control (blue) and case (orange) groups with 90% confidence intervals on the mean shown (shaded areas).

Across both cases and controls, a higher age-related vulnerability score was associated with greater decreases in the Aβ42:Aβ40 ratio and greater increases in pTau, NfL and GFAP concentrations between the pre-pandemic and pandemic assessments (Fig. 3a and Supplementary Table 4). An evaluation of the interaction of the age-related vulnerability score and case–control status found age-related vulnerability-score-dependent increases in pTau-181 (*P* = 0.0170**) and decreases in Aβ42 (*P* = 0.0298*) in those exposed to SARS-CoV-2 relative to controls (Fig. 3b and Supplementary Table 4). The interaction was stronger when an extended set of confounders were modeled (Supplementary Table 5). Age-resolved plots indicate that SARS-CoV-2 cases begin to show greater changes from around 70 years of age (Fig. 4). For an average 75-year-old participant, this model estimated an additional 8.2% increase in pTau-181, a 4.7% decrease in Aβ42 and a 2.3% decrease in the Aβ42:Aβ40 ratio in SARS-CoV-2-positive participants (Methods).

### Effects of other factors on SARS-CoV-2 effects

**Fig. 5:**
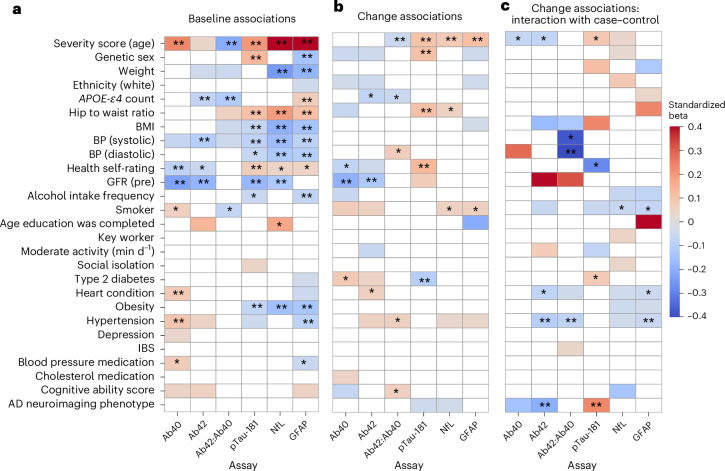
Associations between plasma protein biomarker levels and comorbidities and other factors as determined from regression models across all cases and controls. The shadings show the magnitudes of standardized betas for particular factors, estimated in models with additional variates controlling for age, sex and time interval between assessments. The *P* values correspond to two-sided *t*-tests. All maps masked at |*P* uncorrected| = 0.20. *P uncorrected < 0.05; **FDR significant. **a**, Associations within the baseline assessment data only. The red colors represent positive associations. **b**, Associations of factors with the change in plasma protein biomarker levels across assessments. The red colors represent a positive association of factors with higher levels of protein biomarker in the pandemic assessment session. **c**, Interaction of association from **b** with case–control status (case = +1). The red colors represent a greater positive association in cases.

We assessed similar models to examine the influence of a variety of further comorbidities for AD and other factors on the protein biomarkers (Supplementary Table 1). The plasma protein biomarkers showed a rich set of associations with comorbidities and other factors related to AD (Fig. 5a and Supplementary Table 6). A lower Aβ42:Aβ40 ratio was associated with individuals with an *APOE-ε4* variant (*P* = 0.00021**) and current smokers (*P* = 0.015*). Rates of these variates varied slightly across cases and controls (Table 1) and contributed to a difference in pre-pandemic Aβ42:Aβ40 ratios (0.0747 versus 0.0765, *P* = 0.0145; Supplementary Table 7). These differences were attenuated by regressing *APOE* and smoking status. Differences in baseline measures should not directly affect longitudinal changes after SARS-CoV-2 exposure. This was confirmed when these factors were added to our primary models (Supplementary Table 8).

Several factors were associated with the magnitudes of the longitudinal changes in plasma biomarkers (Fig. 5b, Supplementary Table 9 and Methods). Independent of case–control status, male sex, higher hip-to-waist ratio and poorer health self-rating were associated with greater increases in pTau-181 across assessments (*P* < 0.05**). Type 2 diabetes was associated with reduced changes. A model including these variables indicated that these differences could not account for the differences in protein levels associated with SARS-CoV-2 infection described above (Supplementary Table 10).

A low glomerular filtration rate (GFR) indicates poor kidney function and is known to alter AD biomarker protein levels without increasing disease risk^28^. Here a lower estimated GFR (eGFR; available for *n* = 225 cases) was associated with significantly increased plasma concentrations of protein levels at baseline (Fig. 5a). The eGFR did not show a significant difference between cases and controls (Table 1). A higher GFR was associated with greater reductions in Aβ42 and Aβ40 protein concentrations between assessments, but not with the Aβ42:Aβ40 ratio (Fig. 5b). When modeled as a confounder, GFR had a negligible impact on effect sizes for SARS-CoV-2-associated differences in the Aβ42:Aβ40 ratio (Supplementary Table 11).

### Previous factors associated with plasma biomarker changes

We assessed factors for associations with the SARS-CoV-2-related differences in plasma biomarkers (Fig. 5c and Supplementary Table 12). Previous hypertension was associated with greater reductions in plasma Aβ42 and the Aβ42:Aβ40 ratio in cases (diastolic blood pressure, reduced Aβ42:Aβ40 ratio, *P* = 0.002**; hypertension, reduced Aβ42:Aβ40 ratio, *P* = 0.007*, and reduced Aβ42, *P* = 0.0032**). In previous studies, hypertension has been linked to both more severe COVID-19 symptoms^29^ and greater changes in AD-related fluid biomarkers^30^. While sex, vaccination and *APOE* genotype have been associated with COVID-19 severity^31,32^, our analyses showed no consistent effects for these variables.

An AD brain imaging signature was developed previously from MRI volumetric and cortical thickness measures in the Alzheimer’s Disease Neuroimaging Initiative (ADNI) database^33^, and shown to be associated with a higher incidence of AD and poorer memory and cognitive scores among UK Biobank participants^33^. These AD signature values (after standardization for sex, age and intracranial volume) did not show case–control differences at baseline (*P* = 0.799) or baseline associations with plasma biomarker levels (all *P* > 0.1). However, we found that higher values of the brain imaging signature at baseline were associated with greater increases in pTau-181 (*P* = 0.012**) and greater reductions in Aβ42 (*P* = 0.015**) in cases after SARS-CoV-2 infection than in the matched controls (Fig. 5c and Supplementary Tables 12 and 13).

We also assessed whether baseline plasma levels of a set of inflammatory proteins (Olink) associated with either COVID or AD were associated with plasma biomarker changes (Supplementary Table 14). None differed between cases and controls at the pre-pandemic session (Supplementary Table 15). However, higher baseline levels of macrophage inhibitory factor predicted greater reductions in Aβ protein levels across both cases and controls (Supplementary Table 16). Higher baseline levels of chitinase-3 like-protein-1 (CHI3L1, or YKL-40) were associated with greater Aβ42:Aβ40 ratio reductions in the SARS-CoV-2 group (standardized beta = −0.08, *P* = 0.006**; Supplementary Table 17). CHI3L1 is highly expressed in astrocytes during neuroinflammation and is associated with increased brain amyloid and AD symptoms^34,35^.

### Associations with plasma Olink protein levels

Peripheral inflammation could initiate or potentiate brain β-amyloid pathology after SARS-CoV-2 infection^36^. While in mild cases of COVID-19 inflammatory protein levels have been reported to normalize after infection in the time frame of our study (median time since last SARS-CoV-2 positive test = 68 days)^37^, persistent inflammation, such as that seen in long COVID, could contribute to the changes we observe. We therefore assessed an additional 13 Olink plasma protein markers of inflammation for changes associated with the AD-related biomarkers. TRAIL protein (TNFSF10), an apoptotic TNF cytokine, was significantly reduced after SARS-CoV-2 infection (*P* = 0.002**; Supplementary Table 18). This protein has been reported to be lower in patients with severe COVID^38^ and risk for AD^39^. Those cases who had been hospitalized with COVID-19 showed higher levels of interleukin-6 (*P* = 0.01*) and the long pentraxin PTX3 (*P* = 0.012*) following infection, relative to controls.

Using disease reports from the health records of 47,600 of the UK Biobank participants^39^, the Olink proteins have previously been used to estimate weightings providing individual disease risk scores (ProteinScores; Methods). We were able to estimate 19 of these scores for the pre-pandemic and pandemic assessments to assess whether they were associated with SARS-CoV-2 infection in cases. There were no differences in disease risk scores between cases and controls at baseline, and the incidence of all disease risks (except endometriosis) increased with age. SARS-CoV-2 was associated with increases in disease risk scores for several comorbidities of AD: type 2 diabetes (*P* = 0.015**), chronic obstructive pulmonary disease (COPD; *P* = 0.001**), ischemic stroke (*P* = 0.003**) and heart disease (*P* = 0.013**; Supplementary Table 19). These changes are consistent with recent epidemiological reports of increased incidence of some of these diseases after the pandemic^40^. The AD risk score, weighted heavily to Olink NfL and GFAP levels, did not show a significant increase (*P* = 0.118).

### Associations with neuroimaging, cognitive and health measures

Previous work using the UK Biobank brain imaging data identified changes in brain structure following SARS-CoV-2 infection, linked to age-related vulnerability^9^. We assessed whether these changes were associated with changes in the plasma protein biomarkers. An interaction analysis with the age-dependent vulnerability score showed evidence (*P* = 0.027) for increases in the ADNI-derived AD structural brain phenotype score after SARS-CoV-2 infection in older participants. Increased AD brain signature scores were associated with increased plasma pTau-181 and NfL between visits (*P* = 0.015* and *P* = 0.031, respectively).

Recent work has highlighted that COVID-19 is associated with long-lasting impairments in multiple cognitive domains^10^. We evaluated a measure of general cognitive ability derived from UK Biobank cognitive tests (Methods)^41^. SARS-CoV-2 infection was associated with an additional 1.99% reduction in general cognitive ability over controls, equivalent to almost 2 years of age-related decline (1.16% yr^−1^, *P* = 0.029). These changes were not strongly correlated with the Aβ42:Aβ40 ratio, which can change years before the cognitive changes, but were associated with age-related vulnerability-related changes in Aβ42 (*P* = 0.037) and pTau-181 (*P* = 0.011*).

Participants were asked to rate their overall health between poor and excellent. There were no baseline differences between cases and controls. However, cases showed greater deterioration in their overall health ratings (2.39%, *P* = 0.007*). While previous health self-ratings affected pTau-181 (above), changes in general health self-ratings were not specifically associated with plasma levels of the biomarkers.

## Discussion

SARS-CoV-2 infections can have profound and long-lasting effects on human health. Persistently raised levels of illness and mortality have been reported since the pandemic^31,40^. However, potential effects of mild-to-moderate COVID-19 on risks for late-life dementia have not been well explored. While the observational nature of our study precludes definitive causal inferences, our results provide new evidence that mild-to-moderate SARS-CoV-2 infection may initiate or accelerate brain β-amyloid pathology and suggest a possible increased risk of future AD after infection.

We observed a mean additional reduction in plasma Aβ42:Aβ40 ratio after SARS-CoV-2 infection equivalent to 4 years of aging or 60% of the effect size of inheriting a single *APOE-ε4* allele. Lower Aβ42:Aβ40 ratios are one of the earliest detectable biomarker changes in preclinical stages of AD^16–18^. Participants with greater previous vulnerability showed larger effect sizes and additional decreases in plasma Aβ42 and increases in pTau-181. These changes were associated with an increased brain MRI AD signature; small, but measurably greater, cognitive deficits; and decrements in measures of general health quality. Aβ42 and pTau have consistently been associated with presymptomatic β-amyloid accumulation in AD^16,18,19,42^. SARS-CoV-2 is not neurotrophic^8^, but the infection is associated with systemic inflammatory activation^13^, which can be prolonged even in mild-to-moderate cases of SARS-CoV-2 (ref. ^43^). Associations between peripheral inflammatory events and dementia have been explored in numerous studies^5,6,44,45^. Preclinical studies of peripheral inflammatory challenges have found evidence for brain microglial activation and impaired microglial clearance of β-amyloid and the progression of tau pathology^6,46,47^. Systemic infection could induce CNS inflammation by the trafficking of activated immune cells, infection of vascular endothelial cells or a breakdown of the blood–brain barrier to allow pro-inflammatory plasma proteins into the CNS^36,46^. CNS inflammation can increase the expression of IFITM3 (ref. ^48^), leading to increased Aβ production. IFITM3 variants have been linked to COVID-19 mortality^49^.

An alternative to this immunological mechanism for the biomarker changes could be amyloid deposits linked to premorbid hypoxic or ischemic brain damage and upregulation of BACE1 gene expression, which have been identified in patients who died after severe COVID-19 (ref. ^50^). However, the predominantly mild-to-moderate infections studied here would not have been associated with severe hypoxia.

Increases in circulating NfL and GFAP have been reported with AD, mild cognitive impairment and acute COVID^15,51,52^. However, we did not find increased levels in either following SARS-CoV-2 infection. NfL and GFAP are markers of neural injury and astroglial activation, respectively; increased plasma levels are observed in later stages of AD progression^17,18^, but we expect that few of the participants here have advanced AD pathology. Raised plasma NFL and GFAP concentrations have been observed in patients with COVID in acute settings, but they return to control levels within 6 months unless neurocognitive symptoms persist^37,52,53^.

Aβ and pTau can be affected by other conditions^54–56^. Hepatic dysfunction can raise plasma Aβ and Aβ42:Aβ40 ratios^56^. However, we did not find Olink protein signatures suggesting hepatic dysfunction and SARS-CoV-2 reduced, rather than increased, Aβ42:Aβ40 ratios. Kidney function also can affect plasma protein levels. We identified associations between eGFR and protein levels in both cases and controls, but these did not confound our primary results. Increased plasma pTau-181 is associated with peripheral and central nerve pathology in amyotrophic lateral sclerosis and spinal muscular atrophy^55^. However, we believe that the association of both increased plasma pTau-181 and decreased plasma Aβ42:Aβ40 ratios with SARS-CoV-2 infection most probably reflects β-amyloid brain pathology.

Using Olink plasma-protein-based risk scores, we found evidence for greater predicted risks for diabetes, ischemic stroke and other conditions in participants under the age of 73 (ref. ^39^). This is consistent with emerging post-COVID-19 epidemiology data^40^ and proteomic studies of COVID-19 patients hospitalized with acute symptoms^24^. The changes may be due to common processes initiated with SARS-CoV-2 infection. We found limited evidence for comorbidities directly confounding our biomarker observations. The increased risk of many diseases with SARS-CoV-2 infection raises the possibility that SARS-CoV-2 (and other infections) may accelerate both body and brain aging^57^.

Our study has limitations. Its observational nature identifies associations but precludes causal inferences. Cases were identified from health records and may under-sample asymptomatic cases who were less likely to be tested. Lateral-flow antibody tests (used to identify 161 cases) can report false-positive rates but were bolstered by a follow-up test. Limited symptom information restricted our investigation of the role of the clinical severity of infections. Previous differences in case and control populations could contribute to observed differences. Specifically, individuals with preexisting AD-related pathology have higher rates of SARS-CoV-2 infection^32,58^ and could be overrepresented in the case group. The many health-related variables in UK Biobank enabled us to assess this concern. Case and control groups were well matched by most measures (including cognitive scores, inflammation markers and disease risks). APOE variants did not differ across groups, despite being linked to severe COVID-19 in the broader UK Biobank cohort^32^. There were some differences in infection-risk-related variables including body mass, activity levels and hypertension rates, but these (and other AD comorbidities) were not correlated with longitudinal change in the plasma biomarkers nor did they affect results when modeled as confounders. Nevertheless, it remains possible that these factors, combined with unmeasured susceptibility factors, could be responsible for the observed differences in plasma biomarker levels. Recent studies show that pTau-217 has greater sensitivity to disease progression and amyloid status than pTau-181 (refs. ^59,60^). The pTau-217 assays were not available during the development of our study, and we would expect improved sensitivity if this assay were used in the future. Validation of the associations that we observed in an independent cohort is needed. Generalization of our results to the wider population must be done with care: the UK Biobank sample population is not representative of the general population by many measures, including ethnicity and wealth^61^.

In conclusion, our study found plasma biomarker changes related to higher brain β-amyloid pathology in association with enhanced brain imaging signatures of AD after SARS-CoV-2 infection. While the study does not establish a causal link between infection and AD, along with earlier studies suggesting increased rates of dementia diagnosis following COVID-19 (ref. ^12^), it suggests that the incidence of AD could increase following the COVID-19 pandemic. Importantly, the relationships described here may not be specific for SARS-CoV-2 and reflect more general effects of infections on the aging brain^46^. Prevention of SARS-CoV-2 infection (and other systemic infections) therefore should be considered in developing risk factor modification strategies for AD in later life.

## Methods

### Inclusion and ethics

The UK Biobank is approved by the North West Multi-Centre Research Ethics Committee to obtain and share data and samples from volunteer participants. Written informed consent was obtained from all participants (https://www.ukbiobank.ac.uk/ethics/). The UK Biobank has an ethics and governance committee, a framework with a wide remit (https://www.ukbiobank.ac.uk/learn-more-about-uk-biobank/governance/ethics-advisory-committee) and an active Participant Advisory Group (https://www.ukbiobank.ac.uk/explore-your-participation/stay-involved). The Participant Advisory Group has been consulted in the initial design and further extensions of protocols, including the use of blood samples.

### Design

This study analyzes Simoa ultrasensitive assays of dementia-related proteins taken from the blood plasma of participants of the UK Biobank COVID-19 repeat imaging study^9^. This study identified case and control participants from those who had taken part in the UK Biobank imaging enhancement^62^, which comprises a comprehensive imaging assessment at one of four dedicated sites. Over 40,000 participants had been assessed before the COVID-19 pandemic. Inclusion criteria and other details are described in https://biobank.ndph.ox.ac.uk/showcase/showcase/docs/casecontrol_covidimaging.pdf.

Participants were identified as being SARS-CoV-2 positive by the UK Biobank if (1) there were positive diagnostic antigen tests identified in the linkage to health-related records, (2) COVID-19 was reported in their primary care data or hospital records or (3) a home-based lateral-flow antibody test provided by the UK Biobank was positive and confirmed by an additional follow-up test^63^ (Fortress Fast COVID-19 Home Test, Fortress Diagnostics and ABC-19TM Rapid Test, Abingdon Health). Vaccinated participants positive to the antibody test were given an additional high-affinity blood-based test (Thriva Coronavirus Antibody Test, Thriva). Both diagnostic antigen test and GP and hospital records were accompanied by data permitting the estimation of the date of COVID-19 infection.

Overall, Simoa biomarker measurements from 626 cases and their matched controls were available. Each participant had biomarker measurements from blood samples taken longitudinally from the two imaging assessment sessions (pre- and post-pandemic). Complete pairs of these data were available from 600 cases, and from the 600, their matched controls had complete data. For the 626 cases, SARS-CoV-2 infection was determined from mixed sources: 358 had a GP’s diagnosis of COVID-19, 438 had a record of a positive diagnostic antigen test before the assessment and 467 returned positive on home-based antibody lateral-flow test kits (Fig. 1b). A total of 24 participants had SARS-CoV-2 infection determined during a period of hospitalization, for which 20 had COVID-19 listed as a primary cause. Two participants were excluded for having a previous dementia diagnosis (vascular dementia), identified from hospital inpatient records. Of 289 cases for whom vaccination status could be determined, 40 had data indicating they had been vaccinated before their first positive COVID test.

Control participants were selected as those who had negative antibody test results, and/or no other record of confirmed or suspected COVID-19 from available data. Control participants were matched to positive cases based on five characteristics: sex, ethnicity (non-white or white), date of birth (±6 months), the location of the first imaging assessment and the date of this assessment (±6 months). Further details of case and control identification are provided in the above link and ref. ^9^.

### Proteomic assay acquisition and preprocessing

Aβ40, Aβ42, NfL and GFAP concentrations were measured using the Simoa Human Neurology 4-Plex E assay (Quanterix). The Simoa pTau-181 Advantage Kit was used to measure pTau-181 concentration. All measurements were made according to the manufacturer’s instructions at the University College London UK DRI Fluid Biomarker Laboratory on an HD-X instrument (Quanterix) with one round of experiments and a single batch of reagents. The instrument uses custom algorithms for calibration and quality control (QC). Internal QC samples consisted of pooled plasma and four were run on each plate. Technicians were blinded to the participant case–control status and from which assessment visit a sample was taken. Measurements were all above the limit of detection and coefficients of variation within analytes were below 10%.

Anonymized data from 1,256 participants were integrated with phenotype data via a UK Biobank key. The Simoa proteomic data were Category 163. Four participants were excluded because data were available for only one of the two imaging sessions. pTau-181, NfL and GFAP markers showed positively skewed distributions and were log normalized. For each biomarker, outliers were identified as measurements more than eight times the median absolute deviation and excluded from the analysis. PCR plate differences were also regressed out from the data. There was no significant imbalance of outliers or PCR plates across cases and controls. Cases and controls were matched according to UK Biobank field 4100 with 624 case–control pairs identified. A total of 1,220 participants and 600 matched case–control pairs had complete proteomic data. All analyses were performed on matched data, except (as noted) when key covariates of interest were missing from a set of participants. The Simoa Neurology assays showed patterns of covariance across participants closely matching previous reports (Fig. 5)^64^.

### Olink proteomics

We used Olink Explore proteomic data from the Pharma Proteomics Project, which assayed samples from 54,219 UK Biobank participants across the different assessment sessions^26^. Available data from the COVID-19 imaging assessment sessions for our case–control matched pairs comprised 436 matched participant pairs for which 1,474 proteins from Cardiometabolic, Inflammation, Neurology and Oncology Olink panels were assayed. The Olink 3072 Explore platform extension data were not available for these COVID-19 case–control matched assessment visits. Available Olink proteins included NFL and GFAP, which were also profiled in the Simoa assays. These markers were positively correlated to their Simoa counterparts (Pearson’s *r* = 0.70 and 0.53, respectively, in pre-pandemic measurements).

The Olink panel included cystatin C, which permitted estimation of the GFR (eGFR) in the imaging assessment sessions using the Chronic Kidney Disease Epidemiology Collaboration cystatin C equation^65^. We first assessed how well protein levels measured by OLINK related to serum cystatin C measurements made separately and included in the UK Biobank core blood biochemistry panel data. The latter were derived from the Siemens Advia 1800 platform protein and available only for the initial UK Biobank assessment session. Olink cystatin C measures in our study baseline plasma samples and those from earlier plasma samples acquired on the same participants showed a Pearson’s correlation of 0.84, which was used for appropriate rescaling of the Olink data to enable GFR estimation from the Olink data.

We assessed 13 inflammatory proteins identified from a literature search of associations of inflammatory proteins in blood or CSF with COVID and/or AD (Supplementary Table 14). The inflammatory proteins and risk scores were analyzed for baseline case–control differences and associations with AD protein change using the statistical models outlined below.

We also investigated whether recently defined multivariate plasma protein concentration changes associated with disease risk (ProteinScores) derived from Olink Explore plasma protein biomarkers in 47,600 UK Biobank participants^39^ provided evidence for increased systemic chronic disease risks following SARS-CoV-2 infection. These scores were derived using penalized Cox elastic net regression, which linked the protein levels of some samples in the initial UK Biobank assessment session to over 16 years of primary and secondary care NHS records, disclosing 21 incident outcomes. Being penalized regression, each score comprises weights from 10 to 20 Olink proteins, the selection of which was validated using hold-out data from the UK Biobank. As these risk scores were derived and validated on participants below the age of 73 years, we limited our analysis to this subset of our case–control cohort (that is, the 353 cases and 353 matched controls under the age of 73).

We generated these risk scores for protein profile data from the COVID-19 imaging assessment sessions. The data were rank-base inverse normal transformed and rescaled to match the processing applied in the generation of the ProteinScores. Disease weight scores were downloaded from the publication website and applied to the data^39^.

### Phenotypic data processing

Primary data for the study came from the two imaging assessment sessions. Genetic and phenotypic data, including imaging and health records, were available for all participants who took part in the COVID-19 reimaging project. Symptomology analyses used data from the UK Biobank COVID-19 Serology Study^66^. Data were parsed and cleaned using the FMRIB UK Biobank Normalisation, Parsing and Cleaning Kit, and integrated with other data modalities into a combined Python pandas data frame.

### Genetics

*APOE* variant status was extracted from UK Biobank subject genotyping using PLINK-2.0 (ref. ^67^). We extracted variants at single nucleotide polymorphisms rs429358 and rs7412 to identify participants’ *APOE* status. Data were available for 1,027 participants with 5 genotypes: ε3ε3 (*n* = 630), ε3ε4 (*n* = 235), ε2ε3 (*n* = 135), ε4ε4 (*n* = 20) and ε2ε2 (*n* = 7). We used the ε3ε3 genotype as reference when one was required. Additional AD- and COVID-19-associated single nucleotide polymorphisms in the UK Biobank dataset were sequenced directly or imputed.

### Imaging data and neuroimaging AD phenotype score

To avoid the multiple testing challenge of large numbers of neuroimaging measures and provide a measure recognized to be relevant to AD, we estimated a structural brain data AD phenotype score for each participant at each assessment session using structural-brain-imaging-derived measures made available through the UK Biobank Showcase^68,69^. The AD phenotype score, developed on the ADNI dataset, reflects the prediction of a Bayesian machine learning neural that a participant has AD from 155 FreeSurfer cortical volume and thickness measures^33^. Monte Carlo dropout was used to approximate Bayesian inference within a two-layer neural network. This phenotype model was trained on 736 individuals (331 AD) from the ADNI database, and validated on 5,209 participants from the National Alzheimer’s Coordinating Centre and 37,104 participants in the UK Biobank^33^, where predicted at-risk participants showed cognitive profiles representative of AD.

Neuroimaging measures contributing to the AD phenotype were processed using the same steps used for the generation of the model. Each cortical volume and thickness measure was cleaned by removing outliers more extreme than eight times the median absolute deviation from the median (across both pre-pandemic and pandemic assessment visits). The measures were then de-confounded for age, estimated total intracranial volume and sex, and rescaled using normalization statistics from the ADNI training set. The AD phenotype was then estimated from these data by applying model weights derived from the ADNI dataset^33^. Software to generate neuroimaging signatures, comprising a version-controlled conda environment, was downloaded from https://github.com/tjiagoM/adni_phenotypes. PyTorch (v1.6.0) was used to apply the trained model (https://wandb.ai/tjiagom/adni_phenotypes/runs/2cxy59fk) to UK Biobank data.

### Cognitive scores

The UK Biobank includes a series of standard cognitive tests, but they can be individually unreliable and do not together correspond to established tests for general or dementia-related cognitive scoring. A procedure to estimate a measure of general cognitive ability from the UK Biobank data has previously been developed using an external validation dataset^41^. We used the resulting score weights to calculate an overall score for cognitive ability for participants in each assessment visit.

### Statistical modeling

We assessed longitudinal change in proteomic biomarkers and the effect of SARS-CoV-2 on this change using linear models that modeled protein levels in the pandemic assessment samples in terms of the pre-pandemic protein concentrations, the time between assessment visits, designation as a case or control, and potential confounders including age and baseline differences in genetic backgrounds, sex, health status or social risk factors for COVID between cases and controls^9^. We approached this in a staged fashion that allowed the impact of confounder covariates (some which were available only in a proportion of subjects) to be understood. Our basic model included covariates for sex, age and the interval between assessments:

$$\begin{array}{l}{\rm{Protein}}\_{\rm{post}}={\rm{Protein}}\_{\rm{pre}}+{\rm{Interval}}\_{\rm{between}}\_{\rm{assessments}}\\\qquad\qquad\quad\quad\;+\,{\rm{Interval}}\_{\rm{between}}\_{{\rm{assessments}}}^{2}+{{\text{Case}}-{\text{control}}}\\\qquad\qquad\quad\quad\;+\,{\rm{Genetic}}\_{\rm{sex}}+{\rm{Age}}\_{\rm{post}}\end{array}$$

The extended models included further variables known or expected to change over the assessment period and with COVID-19 (Supplementary Table 1). Some variables presented considerable missing data, such that the extended models had reduced power.

As the impact of SARS-CoV-2 infection is expected to vary according to certain characteristics and vulnerabilities, we fit further models including interaction terms modeling the interaction of SARS-CoV-2 status with characteristics including *APOE* status, hospitalization and pre-pandemic neuroimaging AD score, to determine whether these factors modified the effect of SARS-CoV-2 on protein levels. Age is an important vulnerability factor. Past modeling studies of brain structural changes associated with COVID-19 have used a score of age-dependent vulnerability as an interaction term to account for the greater vulnerability of the brain with age^9^. An exponential age-dependent vulnerability score term (10^Age×0.0524−3.27^) was derived from the modeling of the observed relationship between age and COVID-19 neurological symptom severity (Fig. 4c)^27^. This score was calculated for both cases and controls, so that general aging effects following this estimated trajectory of vulnerability were not confounding assessments. All interaction models included both the original case–control variable and the original vulnerability score variable in addition to the interaction between these variables, along with additional covariates used in the basic model.

In addition to protein levels, we modeled several additional outcome measures: disease risk scores, a general cognitive ability score, a neuroimaging AD score and reported level of general health. These outcomes were modeled in the same manner as the protein levels above. To assess whether changes in these outcomes were associated with changes in protein scores, additional models were assessed, which included as predictors the pre-pandemic levels of proteins and their change across assessment visits. Linear models were also used to assess relationships between protein levels and UK Biobank variables at baseline. Age, sex and interval between assessments were used as confounder variables in these models as above.

The models were fit using ordinary least squares and tested model parameters using *t*-tests. As we had strong hypotheses regarding the expected direction of SARS-CoV-2-induced change in AD protein levels based on previous literature, we used one-sided tests. Benjamini–Hochberg FDR with alpha = 0.05 was used to control for statistical testing of multiple hypotheses across proteins and/or covariates.

### Reporting summary

Further information on research design is available in the Nature Portfolio Reporting Summary linked to this article.

## Online content

Any methods, additional references, Nature Portfolio reporting summaries, source data, extended data, supplementary information, acknowledgements, peer review information; details of author contributions and competing interests; and statements of data and code availability are available at 10.1038/s41591-024-03426-4.

## Supplementary information

Supplementary Information Supplementary Tables 1 and 14, captions for Supplementary Tables 1–20 and Bibliography.

Reporting Summary

Supplementary Tables Supplementary Tables 2–13 and 15–20.

## Data Availability

All data are available upon application to the UK Biobank. UK Biobank Access Policy and Procedures are available from https://www.ukbiobank.ac.uk/enable-your-research. The primary plasma protein data are available under Category Ids 163 (Simoa) and 1839 (Olink). Data fields for other variables are listed in Supplementary Table 1.

## Code availability

Code for primary analyses is available via GitHub at https://github.com/eduff/BIACOB.
