# Supplementary material for: Plasma proteomic evidence for increased β-amyloid pathology after SARS-CoV-2 infection
Source: Nat Med. 2025 Jan 30;31(3):797–806. doi: 10.1038/s41591-024-03426-4 (PMC11922756; doi:10.1038/s41591-024-03426-4)
Supplement: Supplementary file 1 — Supplementary Tables 1 and 14, captions for Supplementary Tables 1–20 and Bibliography. [file 41591_2024_3426_MOESM1_ESM.pdf]

nature medicine

Supplementary information https://doi.org/10.1038/s41591-024-03426-4

Plasma proteomic evidence for increased β-amyloid pathology after SARS-CoV-2 infection

In the format provided by the authors and unedited

Supplementary Information

Tables not shown are provided in the accompanying Excel spreadsheet.

CLASS	COVARIATE	UKB DATA-FIELD ID(S)	CASE/CONTROL COMPARISON	BASIC MODEL	COMORBIDITY MODELS
DEMOGRAPHICS	*Age*	34,52,53	X	X	X
	Sex	31	X	X	X
	Height (cm)	50	X		
	Weight (kg)	21002	X		X
	Ethnicity (White)	21000	X		X
ASSESSMENT INFORMATION	*Date of assessment/ interval*	53	X	X	X
GENETICS	*APOE status*	Genotyping	X		X
HEALTH MEASURES	*Body Mass Index*	21001	X		X
	Hip/Waist Ratio	48,49	X		X
	Blood Pressure (Diastolic, Systolic)	4079,4080	X		X
	Hand grip strength	46,47	X		
	Chest Wheeze	2316	X		
	Est. Glomerular Filtration Rate	Olink: P_CST3	X		X
	General Health	2178	X		X
LIFE STATUS	*Alcohol Intake Freq*	1558	X		X
	Smoking status	1239	X		X
	Years of education	845	X		X
	Deprivation	26410	X		
	Income	738	X		
	Household Size	709	X		X
	Employment status	6142	X		
	Key Worker Status	28063	X		
	Activity levels (min/day)	894,914	X		X
	Isolation (social visits)	1031,2110	X		X
COMORBIDITIES	*Type II Diabetes*	26206	X		X
	Heart Condition	20002	X		X
	Obesity	21001	X		X
	Hypertension	20002	X		X
	Depression	20002	X		X
	Irritable Bowel Syndrome	20002	X		X
	COPD	20002	X		X
	Emphysema	20002	X		X
	Renal conditions	20002	X		X

MEDICATION	*Blood pressure medication*	6153	X		
	Cholesterol medication	6153	X		
	Diabetes medication	6153	X		
	COVID vaccination	Health Records	X		
COGNITION	*General Cognitive Ability score*	Cognitive test fields	X		X
NEUROIMAGING	*AD imaging phenotype*	FreeSurfer IDPs	X		X

Supplementary table 1. UK Biobank Covariates used in analyses. BMI: Body Mass Index; AD: Alzheimer's Disease, PRS: polygenic risk score. COPD - Chronic obstructive pulmonary disease

Supplementary table 2. Parameter fits for linear model predicting proteomic levels in pandemic assessment session. Table shows model fits and p-values associated with case / control status (case=+1), age, sex (male=+1), the time interval between sessions (days), and pre-pandemic protein level. Beta estimates have been standardised. NfL – neurofilament light; GFAP (**) indicates significant, FDR corrected by column, alpha=0.05.

Supplementary table 3. Parameter fits for linear model predicting proteomic levels in pandemic assessment session with an extended set of confound variables. Beta estimates have been standardised. NfL – neurofilament light; GFAP (**) indicates significant, FDR corrected by column, alpha=0.05.

Supplementary table 4. Parameter fits for linear model predicting proteomic levels in pandemic assessment session with additional terms modelling age-related vulnerability and its interaction with case/control status. Beta estimates have been standardised. NfL – neurofilament light; GFAP (**) indicates significant, FDR corrected by column, alpha=0.05.

Supplementary table 5. Parameter fits for linear model predicting proteomic levels in pandemic assessment session with additional terms modelling age-related vulnerability and its interaction with case/control status, and an extended set of confound variables. Beta estimates have been standardised. NfL – neurofilament light; GFAP (**) indicates significant, FDR corrected by column, alpha=0.05.

Supplementary table 6. Associations of protein biomarkers with UK Biobank variables at pre-pandemic assessment visit. Associations were determined from linear models including age and sex covariates. (*) p<0.05 uncorrected (**) p<0.05 FDR correction (alpha = 0.05).

Supplementary table 7. Baseline protein level statistics for cases and controls. P-values reflect paired t-tests.

Supplementary table 8. Parameter fits for linear model predicting proteomic levels in pandemic assessment session with additional terms modelling covariates APOE variant status, smoking status, Hip/Waist ratio and Diabetes. Beta estimates have been standardised. NfL – neurofilament light; GFAP (**) indicates significant, FDR corrected by column, alpha=0.05.

Supplementary table 9. Associations of UK Biobank variables (measured at pre-pandemic assessment) with the change in protein biomarkers across assessments (post-pandemic – pre-pandemic). Associations were determined from linear models of protein level change including terms for age, sex, and interval between assessments. (*) p<0.05 uncorrected (**) p<0.05 FDR correction (alpha = 0.05).

Supplementary table 10. Parameter fits for linear model predicting proteomic levels in pandemic assessment session with additional terms modelling covariates APOE variant status, smoking status, Hip/Waist ratio and Diabetes. Beta estimates have been standardised. NfL – neurofilament light; GFAP (**) indicates significant, FDR corrected by column, alpha=0.05.

Supplementary table 11. Parameter fits for linear model predicting proteomic levels in pandemic assessment session with additional term modelling potential confound Glomerular Filtration Rate (GFR). GFR was available in around half of participants. Beta estimates have been standardised. NfL – neurofilament light; GFAP (**) indicates significant, FDR corrected by column, alpha=0.05.

Supplementary table 12. Parameter fits for interaction term with case-control status (case=+1) and associations between UK Biobank variables at baseline with protein biomarker change. These terms identify UK Biobank variables associated with the protein biomarkers in SARS-CoV-2 exposure specific manner. (*) p<0.05 uncorrected (**) p<0.05 FDR correction (alpha = 0.05).

Supplementary table 13. AD protein level change model fits for models including terms modelling AD neuroimaging phenotype at baseline. Associations were determined from linear models including age and sex covariates. (*) p<0.05 uncorrected (**) p<0.05 FDR correction (alpha = 0.05).

OLINK PROTEIN	COVID	AD
TUMOUR NECROSIS FACTOR ALPHA (TNF-Α)	X[1]	X[2]
TNF SUPERFAMILY MEMBER 10 (TRAIL)	X[1]	X[3]
INTERLEUKIN(IL)-1Α	X[4]	X[5]
IL-6	X[1,6]	X[5]
IL-12		X[7]
IL-18	X[4]	X[8]
IFN-B	X[6]	X[3,5]
INTERFERON GAMMA (IFNG)	X[6]	X[8]
COMPLEMENT C1QA		X[9]
COMPLEMENT C4BPB		X[9]
MACROPHAGE INHIBITORY FACTOR (MIF)	X[10]	X[9,11]
CHITINASE-3 LIKE-PROTEIN-1 (CHI3L1) - YKL-40		X[3]
PENTRAXIN 3 (PTX3)	X[6]	
CALCITONIN (CALCA)	X[1]	

Supplementary table 14. Inflammatory proteins available from Olink panel identified as associated with COVID and/or Alzheimer's Disease. X indicates reports of an association with the disease.

Supplementary table 15 Baseline Case-Control differences in Olink Inflammatory Proteins. P-values reflect paired t-tests.

Supplementary table 16. Associations of Olink Inflammatory Protein levels (measured at baseline) with the change in AD protein biomarkers across assessments (across cases and controls). Associations were determined from linear models including terms for age, sex, and interval between assessments. (*) p<0.05 uncorrected (**) p<0.05 FDR correction (alpha = 0.05).

Supplementary table 17. Associations of an interaction term between Case/Control status and Olink Inflammatory Protein levels (measured at baseline) with protein biomarker change. These identify Olink Inflammatory Proteins associated with the AD protein biomarkers in a SARS-CoV-2 exposure specific manner. Associations were determined from linear models including terms for age, sex, and interval between assessments. (*) p<0.05 uncorrected (**) p<0.05 FDR correction (alpha = 0.05).

Supplementary table 18. Parameter fits for linear model predicting Olink Inflammatory Protein levels in pandemic assessment session. Beta estimates have been standardised. (**) indicates significant, FDR corrected by column, alpha=0.05.

Supplementary table 19. SARS-CoV-2 related increases in Olink ProteinScore disease risk scores. Plots show SARS-CoV-2 effect weighting parameter in model of change of scores between assessment visits. Due to the ProteinScore disease risk training data, results are from participants <73 years. (*) reflects FDR corrected significance, alpha=0.05.

Supplementary table 20. Correlations between Simoa Ultrasensitive neurology assays. Diagonal reflects correlations of protein levels between pre-pandemic and pandemic assessment visits.

Bibliography

1. Byeon, S. K. *et al.* Development of a multiomics model for identification of predictive biomarkers for COVID-19 severity: a retrospective cohort study. *Lancet Digit Health* **4**, e632–e645 (2022).
2. Walker, K. A. *et al.* The role of peripheral inflammatory insults in Alzheimer's disease: a review and research roadmap. *Molecular Neurodegeneration* **18**, 37 (2023).
3. Klyucherev, T. O. *et al.* Advances in the development of new biomarkers for Alzheimer's disease. *Transl Neurodegener* **11**, 25 (2022).
4. Makaremi, S. *et al.* The role of IL-1 family of cytokines and receptors in pathogenesis of COVID-19. *Inflamm. Res.* **71**, 923–947 (2022).
5. Dursun, E. *et al.* The interleukin 1 alpha, interleukin 1 beta, interleukin 6 and alpha-2-macroglobulin serum levels in patients with early or late onset Alzheimer's disease, mild cognitive impairment or Parkinson's disease. *Journal of Neuroimmunology* **283**, 50–57 (2015).

6. Phetsouphanh, C. *et al.* Immunological dysfunction persists for 8 months following initial mild-to-moderate SARS-CoV-2 infection. *Nat Immunol* **23**, 210–216 (2022).
7. Yang, H.-S. *et al.* Plasma IL-12/IFN-γ axis predicts cognitive trajectories in cognitively unimpaired older adults. *Alzheimers Dement* **18**, 645–653 (2022).
8. Sutinen, E. M., Pirttilä, T., Anderson, G., Salminen, A. & Ojala, J. O. Pro-inflammatory interleukin-18 increases Alzheimer's disease-associated amyloid-β production in human neuron-like cells. *Journal of Neuroinflammation* **9**, 199 (2012).
9. Brosseron, F. *et al.* Soluble TAM receptors sAXL and sTyro3 predict structural and functional protection in Alzheimer's disease. *Neuron* **110**, 1009-1022.e4 (2022).
10. Shin, J. J. *et al.* MIF is a common genetic determinant of COVID-19 symptomatic infection and severity. *QJM* **116**, 205–212 (2023).
11. Nasiri, E. *et al.* Key role of MIF-related neuroinflammation in neurodegeneration and cognitive impairment in Alzheimer's disease. *Mol Med* **26**, 34 (2020).
