# Supplementary material for: Plasma proteomic evidence for increased β-amyloid pathology after SARS-CoV-2 infection
Source: Nat Med. 2025 Jan 30;31(3):797–806. doi: 10.1038/s41591-024-03426-4 (PMC11922756; doi:10.1038/s41591-024-03426-4)
Supplement: Supplementary file 2 — Reporting Summary [file 41591_2024_3426_MOESM2_ESM.pdf]

nature portfolio

Corresponding author(s): Eugene Duff , Paul Matthews

Last updated by author(s): Nov 14, 2024

Reporting Summary

Nature Portfolio wishes to improve the reproducibility of the work that we publish. This form provides structure for consistency and transparency in reporting. For further information on Nature Portfolio policies, see our Editorial Policies and the Editorial Policy Checklist.

Statistics

For all statistical analyses, confirm that the following items are present in the figure legend, table legend, main text, or Methods section.

n/a	Confirmed	
☐	☒	The exact sample size (*n*) for each experimental group/condition, given as a discrete number and unit of measurement
☐	☒	A statement on whether measurements were taken from distinct samples or whether the same sample was measured repeatedly
☐	☒	The statistical test(s) used AND whether they are one- or two-sided<br>*Only common tests should be described solely by name; describe more complex techniques in the Methods section.*
☐	☒	A description of all covariates tested
☐	☒	A description of any assumptions or corrections, such as tests of normality and adjustment for multiple comparisons
☐	☒	A full description of the statistical parameters including central tendency (e.g. means) or other basic estimates (e.g. regression coefficient) AND variation (e.g. standard deviation) or associated estimates of uncertainty (e.g. confidence intervals)
☐	☒	For null hypothesis testing, the test statistic (e.g. *F*, *t*, *r*) with confidence intervals, effect sizes, degrees of freedom and *P* value noted<br>*Give P values as exact values whenever suitable.*
☒	☐	For Bayesian analysis, information on the choice of priors and Markov chain Monte Carlo settings
☒	☐	For hierarchical and complex designs, identification of the appropriate level for tests and full reporting of outcomes
☐	☒	Estimates of effect sizes (e.g. Cohen's *d*, Pearson's *r*), indicating how they were calculated

Our web collection on statistics for biologists contains articles on many of the points above.

Software and code

Policy information about availability of computer code

Data collection	All measurements were made according to manufacturer's instructions at the University College London UKDRI Fluid Biomarker Lab on an HD-X instrument (Quanterix) with one round of experiments and a single batch of reagents. The instrument uses custom algorithms for calibration and quality control.
Data analysis	Software to generate neuroimaging signatures, comprising a version-controlled conda environment, was downloaded from https://github.com/tjiagoM/adni_phenotypes . PyTorch (v1.6.0) was used to apply the trained model (https://wandb.ai/tjiagom/adni_phenotypes/runs/2cxy59fk) to UK Biobank data. Code for primary analyses are available from https://github.com/eduff/BIACOB.

For manuscripts utilizing custom algorithms or software that are central to the research but not yet described in published literature, software must be made available to editors and reviewers. We strongly encourage code deposition in a community repository (e.g. GitHub). See the Nature Portfolio guidelines for submitting code & software for further information.

Data

Policy information about availability of data

All manuscripts must include a data availability statement. This statement should provide the following information, where applicable:

- Accession codes, unique identifiers, or web links for publicly available datasets
- A description of any restrictions on data availability
- For clinical datasets or third party data, please ensure that the statement adheres to our policy

All data is available upon application from the UK Biobank. UK Biobank Access Policy and Procedures are available from www.ukbiobank.ac.uk/enable-your-research . The primary plasma protein data are available under Category Ids 163 (Simoa) and 1839 (Olink). Covariate data fields are listed in Supplementary Table 1.

Research involving human participants, their data, or biological material

Policy information about studies with human participants or human data. See also policy information about sex, gender (identity/presentation), and sexual orientation and race, ethnicity and racism.

Reporting on sex and gender	The study population included approximately equal numbers of males and females, with sex determined by genetic analysis. Control participant matching included genetic sex. Gender was not considered in this study of viral infection.
Reporting on race, ethnicity, or other socially relevant groupings	The study includes an analysis of self-reported ethnic background, using categories predefined by the UK Biobank. Due to very limited numbers and low power, ethnicity was characterised only by white/non-white classification. UK
Population characteristics	Proteomic data were available from a subset of UK Biobank participants who took part in the COVID repeat imaging study. UK Biobank participants comprise volunteers living in the UK who were 40-69 of age at recruitment. No particular population characteristics were selected for. However, participants differ from eligible non-participants in various ways, including being more likely to be female, having fewer health conditions, and less likely to live in economically deprived areas. See Fry et al Comparison of Sociodemographic and Health-Related Characteristics of UK Biobank Participants With Those of the General Population Am J Epidemiol 2017.
Recruitment	UK Biobank recruited 500,000 people across the country aged between 40 and 69 years from 2006 to 2010 through their GPs. Participants were followed up by mail for recruitment into the imaging and COVID substudies.
Ethics oversight	The UK Biobank is approved by the North West Multi-Centre Research Ethics Committee (MREC) to obtain and share data and samples from volunteer participants. Written informed consent was obtained from all participants (http://www.ukbiobank.ac.uk/ethics/).

Note that full information on the approval of the study protocol must also be provided in the manuscript.

Field-specific reporting

Please select the one below that is the best fit for your research. If you are not sure, read the appropriate sections before making your selection.

☒ Life sciences ☐ Behavioural & social sciences ☐ Ecological, evolutionary & environmental sciences

For a reference copy of the document with all sections, see nature.com/documents/nr-reporting-summary-flat.pdf

Life sciences study design

All studies must disclose on these points even when the disclosure is negative.

Sample size	All available case-control matched COVID repeat imaging participants were assayed for the neurobiomarkers. This dataset is comparable or larger in size to all validation studies for these neurobiomarkers. While anticipated effect sizes for SARS-CoV-2 infection were unknown, previous studies had identified genetic variant effects on these markers in cohorts without individual case-control matching (e.g. Stevenson-Hoare et al Brain 2023).
Data exclusions	Participants with prior dementia diagnosis were excluded
Replication	No comparable replication datasets were available. Statistical analyses used the full data to optimise sensitivity
Randomization	This was an observational prospective case-control study with individually matched controls. Samples were randomized across PCR plates.
Blinding	Sample acquisition and processing was performed in a blinded Data blinding was not possible for statistical analysis as Case group was linked to group-specific meta-data used in all modelling (e.g. source of positive COVID-test; hospitalisation)

Reporting for specific materials, systems and methods

We require information from authors about some types of materials, experimental systems and methods used in many studies. Here, indicate whether each material, system or method listed is relevant to your study. If you are not sure if a list item applies to your research, read the appropriate section before selecting a response.

Materials & experimental systems

n/a	Involved in the study
☒	☐ Antibodies
☒	☐ Eukaryotic cell lines
☒	☐ Palaeontology and archaeology
☒	☐ Animals and other organisms
☒	☐ Clinical data
☒	☐ Dual use research of concern
☒	☐ Plants

Methods

n/a	Involved in the study
☒	☐ ChIP-seq
☒	☐ Flow cytometry
☐	☒ MRI-based neuroimaging

Plants

Seed stocks: n/a

Novel plant genotypes: n/a

Authentication: n/a

Magnetic resonance imaging

Experimental design

Design type: Pre-processed diffusion and structural MRI "Imaging derived phenotypes" (IDPs) provided with the UK Biobank were used for a sub-analysis.

Design specifications: We used IDPs derived from structural and diffusion scans from two UK Biobank imaging assessment sessions, which were typically separated by several years.

Behavioral performance measures: None associated with the MRI data

Acquisition

Imaging type(s): Structural & Diffusion

Field strength: 3

Sequence & imaging parameters: Full details of the imaging parameters from which the IDPs were derived are available in the referenced publications

Area of acquisition: Whole brain

Diffusion MRI: ☒ Used ☐ Not used

Parameters: Full details of the imaging parameters from which the IDPs were derived are available in the referenced publications

Preprocessing

Preprocessing software: The IDPs provided by the UK Biobank were generated by FSL & FreeSurfer

Normalization: Details provided in referenced papers

Normalization template: Details provided in referenced papers

Noise and artifact removal | Details provided in referenced papers

Volume censoring | n/a

Statistical modeling & inference

Model type and settings | Weightings for an AD phenotype score were derived in an independent referenced study. AD phenotype scores were estimated for the UK Biobank IDPs and then analysed in linear models of SARS-CoV-2 effects on protein biomarker levels and the phenotype scores.

Effect(s) tested | Association of imaging phenotype with sARS-CoV-2-positivity and protein biomarkers.

Specify type of analysis: ☐ Whole brain ☒ ROI-based ☐ Both

Anatomical location(s) | UK Biobank IDPs are provided for FreeSurfer Cortical Parcellations regions, which were used to estimate AD phenotype scores.

Statistic type for inference (See Eklund et al. 2016) | Inference was performed on AD-phenotype scores estimated for a particular scan session. t-statistics associated with hypothesis tests on parameters from linear regression models were assessed.

Correction | FDR (α=0.05) across proteins

Models & analysis

n/a	Involved in the study
☒	☐ Functional and/or effective connectivity
☒	☐ Graph analysis
☐	☒ Multivariate modeling or predictive analysis

Multivariate modeling and predictive analysis | "MRI AD Phenotype" is a multivariate score (weightings) associated with AD derived in a previous study. In this study we calculate its value for our participants and test its association with key covariates.
